# Association Between Early Cognitive Impairment and Midterm Functional Outcomes Among Chinese Acute Ischemic Stroke Patients: A Longitudinal Study

**DOI:** 10.3389/fneur.2020.00020

**Published:** 2020-02-26

**Authors:** Juan Li, Jing Wang, Bei Wu, Hanzhang Xu, Xiongfeng Wu, Lanshu Zhou, Benqiang Deng

**Affiliations:** ^1^Naval Military Medical University School of Nursing, Shanghai, China; ^2^Fudan University School of Nursing, Shanghai, China; ^3^New York University Rory Meyers College of Nursing, New York, NY, United States; ^4^Duke University School of Nursing, Durham, NC, United States; ^5^Duke University School of Medicine, Durham, NC, United States; ^6^Stroke Center, Changhai Hospital Affiliated to Naval Military Medical University, Shanghai, China

**Keywords:** cognitive impairment, recovery of function, acute ischemic stroke, MoCA, mRS, BI, longitudinal study

## Abstract

**Background:** Cognitive decline is common after stroke. The influence of early cognitive impairment on midterm functional outcomes among Chinese acute ischemic stroke (AIS) patients has not been fully studied. The aim of the study was to assess the association between early cognitive impairment and midterm functional outcomes among Chinese AIS patients.

**Methods:** A longitudinal survey focusing on Chinese AIS patients was conducted in three stroke centers in Shanghai, China (July to December 2016). A total of 185 eligible patients were interviewed at acute stage and at 1, 3, and 6 months after onset. Patients' functional outcomes were measured by modified Rankin Scale (mRS) and Barthel Index (BI) at each time point. Cognitive function was assessed using Montreal Cognitive Assessment, Changsha version (MoCA-CS), within 7 days after stroke onset. Covariates included patient's demographic characteristics, socioeconomic status, clinical characteristics of stroke, vascular risk factors, receiving rehabilitation after discharge from acute hospital, and recurrence. Generalized linear mixed models and general linear mixed models were applied.

**Results:** The prevalence of cognitive impairment at acute stage of stroke among these patients was 88.1%. The risk of disability (mRS 2–5) of all patients after stroke decreased over time (OR = 0.491, 95% CI = 0.401–0.603). The risk of disability among those with cognitive impairment increased compared with those with normal cognition (OR = 7.384, 95% CI = 1.041–52.407). The BI score of all patients increased over time after controlling for covariates (β = 1.51, *p* < 0.01). The BI score of those with cognitive impairment was lower than that with normal cognition over the follow-up period after controlling for other covariates (β = −8.11, *p* < 0.05).

**Conclusions:** This study showed that early cognitive impairment was associated with higher risk of disability and poor activity of daily living (ADL) among Chinese AIS patients. Further studies are needed to examine the linkage between multi-domain cognitive impairment and long-term disability and ADL among stroke survivors by using neuropsychological test batteries.

## Introduction

Stroke is the second leading cause of death and the third leading cause of disability globally (1). It has significant impact on disability and imposes heavy financial burden on patients, families, and the health-care system (1). China had the highest stroke incidence in the world in 2010 (2). There are more than 70 million stroke patients in China (3, 4). Two million new cases of stroke occur every year, and the incidence is rising at the rate of 8.7% every year (5). Cognitive impairment is common among stroke patients (6). More than two thirds of stroke patients at the acute stage and 69.8–96% patients in the following 3–6 months after onset have cognitive impairment (7–10). Cognitive impairment after stroke is related to poorer clinical outcomes, such as higher rate of disability, institutionalization of long-term care facilities, recurrence of stroke, and mortality (11–13). It increases the cost of care and burden of caregivers and decreases the quality of life of stroke survivors and caregivers (14). The proclamation of the 2015 World Stroke Day was “call to preserve cognitive vitality” (15). It would be important to study the association between cognitive impairment at acute stage and disability and activities of daily living (ADLs) among stroke patients, so appropriate intervention and program can be developed to help in the management of stroke.

Several studies have found that cognitive impairment after stroke was related to poor functional outcomes, but there were some methodological limitations: (I) most of the previous studies reported the functional status or the percentage of functional dependency among stroke patient at some special time points after onset, and few studies examined the trajectories (changes with time) of functional outcome after stroke (14, 16, 17). (II) Previous studies used cross-sectional design and explored the relationship between cognitive impairment and physical function at acute stage and at 3–6 months or 1–3 years after stroke onset (18–21). Few studies had conducted longitudinal research and focused on the association between early cognitive impairment at acute stage of hospitalization and functional outcomes among stroke patients (16). However, the study has not considered the influence of therapeutic regimen (intravenous thrombolysis, and intra-arterial thrombectomy) on functional outcome when exploring the association between cognitive function at acute stage and long-term functional outcome. (III) There was not report about association between early cognitive impairment and functional outcome among Chinese stroke patients. It is unknown whether early cognitive impairment at acute stage of hospitalization is associated with midterm outcomes after controlling premorbid disability, cognitive status, demographic characteristics, stroke severity, and treatment options among Chinese stroke patients.

The aims of this study were (I) to examine the trajectories of functional outcomes from stroke onset to 6 months among Chinese patients with acute ischemic stroke (AIS) and (II) to investigate the association between early cognitive impairment and midterm functional outcomes after controlling for other covariates (sociodemographic and clinical characteristics). To our knowledge, this is the first study that assessed the association between early cognitive impairment and midterm functional recovery in Chinese AIS patients. The findings from this study will provide health professionals with useful information about the management and rehabilitation for stroke patients.

## Materials and Methods

### Study Settings

This study was conducted in three stroke centers of tertiary hospitals in Shanghai, China. These three stroke centers in Shanghai were certified by the National Health Commission of the People's Republic of China or Shanghai Municipal Health Commission, which are representative of stroke centers of Shanghai. The median bed size of these three hospitals as 1,304, and annual ischemic stroke volume of the three stroke centers was 486, which was similar to the national estimates [median bed size 1,000 (600–1,500); median annual ischemic stroke volume 447 (320–804)] (22).

### Patients

We recruited patients with AIS from three major stroke centers between July and December 2016 in Shanghai, China. Inclusion criteria were as follows: (1) diagnosed with AIS by a neurologist or a neurosurgeon on the basis of a focal neurologic deficit and a corresponding infarct on brain magnetic resonance imaging (MRI), (2) admitted to a stroke center within 1 week after stroke symptom onset, (3) age ≥ 18 years, and (4) consent to participate in the study. Exclusion criteria were as follows: (1) consciousness obscurity or loss of consciousness, (2) dementia or mental disease being diagnosed by a doctor prior to stroke onset, (3) having aphasia or severe visual or hearing impairment or paretic right hand, and (4) having a malignant disease with life expectancy of <6 months. The diagnoses of dementia and other mental disorders were obtained through a retrospective chart review. The total number of patients with a final diagnosis of AIS admitted in the respective stroke centers during the enrollment period was 305. Of the 305 patients, 205 were eligible patients, and 194 patients provided informed consent to participate in the study.

### Instruments

#### Modified Rankin Scale

The modified Rankin Scale (mRS) was widely used as a reliable functional abilities scale in clinical trials for stroke patients (23). The mRS measured independence rather than performance of specific tasks with scores that ranged from 0 to 6. Score 0 represented no disability, and 6 represented death. The mRS score of 0–1 indicated excellent outcome of clinical recovery (coding = 0) among stroke patients, whereas mRS score of 2–5 indicated mild to severe disability (coding = 1) (24–28).

#### Barthel Index

The Barthel Index (BI) was used to assess the patient's level of independence in 10 ADLs. The items were related to self-care (feeding, grooming, bathing, dressing, bowel and bladder care, and toilet use) and mobility (ambulation, transfers, and stair climbing). The BI was a continuous variable, and it ranged from 0 to 100 with higher score indicating more independence of ADL (29).

#### Montreal Cognitive Assessment—Changsha Version

The Montreal Cognitive Assessment (MoCA) is an cognitive screening tool and includes the assessment of visuospatial/executive function, naming, memory, attention, language, abstraction delayed recall, and orientation (30). It has good sensitivity and specificity in detecting cognitive impairment (31–35). MoCA score ranged from 0 to 30, with lower score indicating poorer cognition (30). MoCA has been used to screen vascular cognitive impairment for stroke or TIA patients (31–35). The Changsha version of the MoCA (MoCA-CS) is a Chinese version of MoCA, which was modified from the original MoCA (English version) by a research team on the basis of Chinese cultural, linguistic, and population characteristics. The MoCA-CS was tested in a sample of Chinese ischemic cerebrovascular disease patients, and the results showed that it was a reliable and valid measure (36, 37). The optimal cutoff points of MoCA-CS for detecting cognitive impairment were 26/27 (sensitivity 96.1% and specificity 75.6%). MoCA was transferred to a dichotomous variable (1 = cognitive impairment and 0 = normal cognition). Those who had no more than 6-year education add 1 point to the total score (36, 37).

### Outcome Variable

The functional outcomes of AIS patients were measured by mRS and BI.

### Independent Variable

Independent variable was cognitive function assessed by MoCA-CS.

### Covariates

The main covariates were demographic characteristics, socioeconomic status (SES), severity of stroke [National Institutes of Health Stroke Scale (NIHSS), dysphagia, pain, and length of stay] (30, 31), classification of stroke [Oxfordshire Community Stroke Project (OCSP) Classification and Trial of ORG 10172 in Acute Stroke Treatment (TOAST) classification] (32, 33), therapeutic options (intravenous thrombolysis, intra-arterial thrombectomy, and rehabilitation), comorbidities (hypertension, diabetes, dyslipidemia, obstructive sleep apnea syndrome, carotid atherosclerosis, and atrial fibrillation), and risk factors (previous history of stroke, smoking, drinking, premorbid disability, and recurrence). The covariates are listed in Table 1. Inpatient and outpatient rehabilitation included physical therapy, occupational therapy, and speech therapy. Water swallowing test was to evaluate the level of dysphagia. The score ranged from I to V. Score I indicated no dysphagia, whereas scores II–V were positive, which indicated dysphagia (34). The level of pain was assessed by a self-report scale that ranged from 0 to 10, with higher score indicating higher level of pain. Premorbid disability before onset was measured by mRS, and the cutoff points are already discussed in the *Outcome Variable* section.

**Table 1:** The list of covariates.

**Covariates**	**Variable**		**Coding**
Demographic characteristics	Age	Continuous variable	
	Gender	Categorical variable	Male = 1, female = 0
	Ethnicity	Categorical variable	Han = 1, Non-Han = 0
	Marital status	Categorical variable	Married = 1, others = 0
Socioeconomic status (SES)	Education years	Continuous variable	
	Rural/urban residency	Categorical variable	Urban = 1, rural = 0
	Family income	Categorical variable	<50,000 yuan per year = 1, 50,000–100,000 yuan per year = 2, >100,000 yuan per year = 3
Severity of stroke	NIH Stroke Scale (NIHSS) (at admission)	Continuous variable	
	NIHSS (at discharge)	Continuous variable	
	Dysphagia (water swallowing test)	Categorical variable	Yes = 1, no = 0
	Pain	Continuous variable	
	Length of stay	Continuous variable	
Classification of stroke	Oxfordshire Community Stroke Project (OCSP) Classification	Categorical variable	Total anterior circulation infarct (TACI) = 1
			Partial anterior circulation infarct (PACI) = 2
			Posterior circulation infarct (POCI) = 3
			Lacunar circulation infarcts (LACI) = 4
	Trial of ORG 10,172 in Acute Stroke Treatment (TOAST) classification	Categorical variable	Large-artery atherothrombotic (LAA) = 1
			Cardioembolic (CE) = 2
			Small-artery occlusion (SAO) = 3
			Other determined etiology (ODE) = 4
			Undetermined etiology (UDE) = 5
Therapeutic options	Intravenous thrombolysis	Categorical variable	Yes = 1, no = 0
	Intra-arterial thrombectomy	Categorical variable	Yes = 1, no = 0
	Receiving rehabilitation in 1 month after onset	Categorical variable	Yes = 1, no = 0
Comorbidities	Hypertension	Categorical variable	Yes = 1, no = 0
	Diabetes	Categorical variable	Yes = 1, no = 0
	Dyslipidemia	Categorical variable	Yes = 1, no = 0
	Obstructive sleep apnea syndrome	Categorical variable	Yes = 1, no = 0
	Carotid atherosclerosis	Categorical variable	Yes = 1, no = 0
	Atrial fibrillation	Categorical variable	Yes = 1, no = 0
Risk factors	Previous history of stroke	Categorical variable	Yes = 1, no = 0
	Smoking	Categorical variable	Yes = 1, no = 0
	Drinking	Categorical variable	Yes = 1, no = 0
	Premorbid disability	Categorical variable	Yes = 1, no = 0
	Recurrence	Categorical variable	Yes = 1, no = 0

### Procedures

Three trained research assistants consented and recruited eligible patients. Three trained neurologists and three research assistants made an assessment on patients' functional and cognitive status. The research team conducted an in-person interview with the patients and their family members during baseline (during the patients' stay at the hospital) and had telephone interviews with them at 1, 3, and 6 months after patients' onset of stroke. The neurologists administered the MoCA-CS within 7 days after the onset of stroke. The research assistants administered the mRS and BI in person during the baseline and then administered them via telephone at 1, 3, and 6 months after the baseline. Accepting rehabilitation after discharge from acute hospital and recurrence was measured at each follow-up interview. Pain was measured at four time points. All other covariates were measured at baseline.

### Statistical Analysis

Descriptive statistics were used to summarize baseline sociodemographic status and clinical characteristics. Baseline characteristics of patients were reported as mean and standard deviation (SD) or median and interquartile for continuous variables and as numbers and proportion for categorical variables. Differences in the patients' characteristics between three centers were evaluated with analysis of variance (ANOVA) or Kruskal–Wallis *H* test for the continuous variables. We used the χ^2^ or the Fisher exact test for categorical variables (Appendix 1). The score of BI was a continuous variable, whereas the score of mRS was a dichotomous variable. All the categorical variables were transferred to dummy variables. A time variable was also derived to indicate the time of each interview since entering into the study (time = 0th, 1st, 3rd, and 6th month) in relation to the baseline and follow-up. A series of generalized linear mixed models were used to estimate the association between acute cognitive impairment and mRS (time = 0th, 1st, 3rd, and 6th month) over time. Similarly, generalized linear mixed models were used to examine the association between acute cognitive impairment and BI (time = 0th, 1st, 3rd, and 6th month) over time. Firstly, we run the univariate generalized linear mixed model to test the association between independent variables and mRS, and we run the univariate generalized linear mixed model to test the association between independent variables and BI. Then only the statistically significant independent variables (*p* < 0.05) were entered into the multivariate generalized linear mixed models or generalized linear mixed models. The procedure of PROC GLIMMIX and PROC MIXED in SAS Version 9.4 (SAS Institute Inc., Cary, NC) was used to estimate the parameters of the generalized linear mixed models and linear mixed models. For the mRS, (1) model I was a basic model adjusted for time and cognitive impairment; (2) model I plus NIHSS, premorbid disability, and hypertension (model II); and (3) model II plus education and having a caregiver (model III). For the BI, (1) model I was a basic model adjusted for time and cognitive impairment; (2) model I plus NIHSS, receiving rehabilitation 1 month after onset (model II); and (3) model II plus premorbid disability, hypertension, and pain (model III). The significant level was set at *p* < 0.05.

## Results

**Figure 1:**
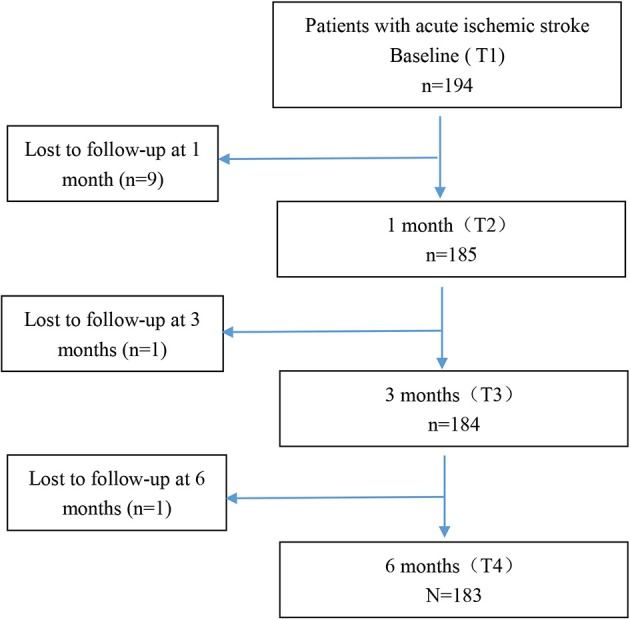
Flowchart of study patients.

Figure 1 demonstrates the flowchart of the study patients. There were 194 patients at baseline and 183 patients at 6 months after onset. One hundred eighty-five patients had more than two time points of data, and these patients were included into the final analysis. Eleven patients were lost to follow-up because our team was not able to reach them owing to inaccurate phone number or refusal to receive phone interview. There were no statistically significant differences on age, gender, education, marital status, NIHSS, mRS, and BI between the patients who were lost to follow-up (*N* = 11) and those who completed the study (*N* = 183). However, the MoCA of the patients who were lost to follow-up was significantly lower than that of those who completed the study (*p* < 0.05). It may be explained that those patients with poor cognition were more likely to discontinue participation in the study owing to severe impairment. Also the differences we observed in this study might be more conservative. The age, NIHSS, TOAST classification, and comorbidities of the patients in our study were consistent with national estimates about mild stroke patients reported in the China National Stroke Registry II (22, 24, 35).

Table 2 presents the baseline sociodemographic and clinical characteristics of the patients. Among 185 patients with AIS, 163 (88.1%) had cognitive impairment at acute stage (22 had normal cognition). Compared with patients with normal cognition, those with cognitive impairment had lower level of education, higher level of stroke severity and pain, longer time of onset to door, and higher proportion of diabetes and drinking (*p* < 0.05 for all).

**Table 2:** Patient sample and characteristics for patients with acute ischemic stroke (*N* = 185).

**Characteristics**	**Normal cognition (*n* = 22)**	**Cognitive impairment (*n* = 163)**	***p*-value**
Age, years; median (IQR)	64 (54–70)	61 (56–65)	0.231
Sex, male (%)	14 (63.6%)	133 (81.6%)	0.05
Ethnicity—Han	22 (100%)	163 (100%)	
Education			0.002^*^
Illiterate	1 (4.5%)	8 (4.9%)	
Elementary school	0 (0)	11 (6.7%)	
Junior school	4 (18.2%)	72 (44.2%)	
High school	8 (36.4%)	54 (33.1%)	
College and university	9 (40.9%)	18 (11.0%)	
Marital status, living spouse (%)	21 (95.5%)	150 (92.0%)	0.568
Residency, urban (%)	20 (90.9%)	150 (92.0%)	0.857
Working, yes (%)	11 (50%)	67 (41.1%)	0.428
NIHSS at admission, median (IQR)	1 (0–2)	2 (1–5)	0.006^*^
NIHSS at discharge, median (IQR)	0 (0–1)	1 (0–3)	0.009^*^
Dysphagia (%)	3 (13.6%)	53 (32.5%)	0.070
Pain, mean (SE)	0.08 (0.07)	0.33 (0.04)	0.003^*^
Length of stay, median (IQR)	9 (7–9.25)	8 (7–9)	0.050
OCSP classification			0.677
TACI	1 (5.6%)	7 (4.6%)	
PACI	2 (27.8%)	63 (41.4%)	
POCI	3 (33.3%)	47 (30.9%0)	
LACI	6 (33.3%)	35 (23.0%)	
TOAST classification			0.737
LAA	8 (36.4%)	77 (47.2%)	
CE	1 (4.5%)	12 (7.4%)	
SAO	11 (50.0%)	57 (35.0%)	
ODE	1 (4.5%)	10 (6.1%)	
UDE	1 (4.5%)	7 (4.3%)	
Intravenous thrombolysis (%)	4 (18.2%)	19 (11.7%)	0.384
Intra-arterial thrombectomy (%)	0 (0)	3 (1.8%)	0.521
Comorbidities			
Hypertension	15 (68.2%)	117 (71.8%)	0.726
Diabetes	4 (18.2%)	67 (41.1%)	0.038^*^
Dyslipidemia	10 (45.5%)	46 (28.2%)	0.099
Obstructive sleep apnea syndrome	0 (0)	2 (1.2%)	0.601
Carotid atherosclerosis	0 (0)	8 (4.9%)	0.288
Atrial fibrillation	1 (4.5%)	12 (7.4%)	0.628
Others	0 (0)	5 (3.1%)	0.405
Previous stroke history (%)	1 (4.5%)	32 (19.6%)	0.083
Smoking (%)	8 (36.4%)	88 (54.0%)	0.120
Drinking (%)	7 (31.8%)	91 (55.8%)	0.034^*^
Premorbid disability	3 (13.6%)	25 (15.7%)	0.800
Rehabilitation in 1 month	3 (13.6%)	8 (4.9%)	0.106
Recurrence in 1 month after onset	0 (0)	4 (2.5%)	0.456
Recurrence in 3 months after onset	0 (0)	4 (2.5%)	0.456
Recurrence in 6 months after onset	0 (0)	1 (0.6%)	0.710

TACI, total anterior circulation infarct; PACI, partial anterior circulation infarct; POCI, posterior circulation infarct; LACI, lacunar circulation infarcts; LAA, large-artery atherothrombotic; CE, cardioembolic; SAO, small-artery occlusion; ODE, other determined etiology; UDE, undetermined etiology; IQR, interquartile range; NIHSS, National Institutes of Health Stroke Scale; OCSP, Oxfordshire Community Stroke Project.

* *means statistically significant*.

**Figure 2:**
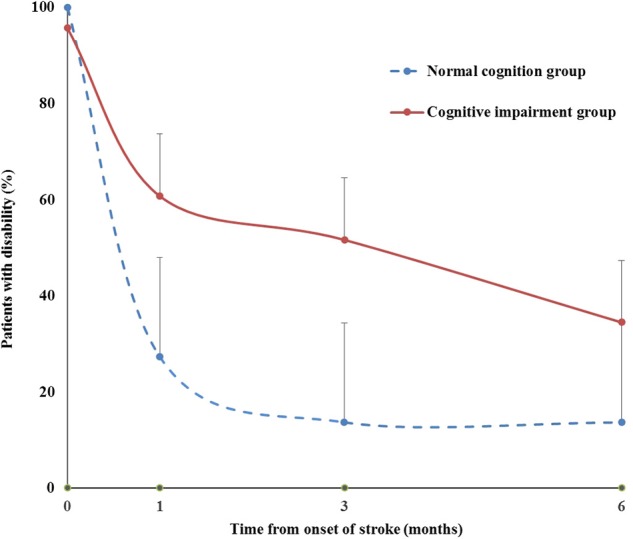
Time course of disability by baseline cognitive function.

Table 3 presents the results from generalized linear mixed models of cognitive impairment and the covariates on disability (mRS 2–5). We found that the likelihood of being disabled decreased over time after stroke onset (OR = 0.491, 95% CI = 0.401–0.603). Cognitive impairment was significantly associated with disability. The risk of disability among those with cognitive impairment increased compared with those with normal cognition (OR = 7.384, 95% CI = 1.041–52.407). Compared with those without premorbid disability, among patients with premorbid disability, the likelihood of being disabled after stroke increased (OR = 16.012, 95% CI = 2.678–95.723). Figure 2 demonstrates the time course of disability by baseline cognitive function. The percentage of disability in cognitive impairment group was higher than that in normal cognition group with time.

**Table 3:** The results from generalized linear mixed models on disability.

**Variables**	**Disability (mRS 2–5)**											
	**Model I**				**Model II**				**Model III**			
	**OR**	**95% CI**		***p***	**OR**	**95% CI**		***p***	**OR**	**95% CI**		***p***
		**Lower**	**Upper**			**Lower**	**Upper**			**Lower**	**Upper**	
Intercept				0.9160				0.2702				0.5672
Time	0.551	0.463	0.655	<0.0001	0.511	0.421	0.620	<0.0001	0.491	0.401	0.603	<0.0001
Cognitive impairment	16.528	3.536	77.249	0.0004	12.655	2.563	62.471	0.0020	7.384	1.041	52.407	0.0456
NIHSS (at discharge)					1.161	0.835	1.613	0.3730	1.133	0.798	1.607	0.4834
premorbid disability					13.692	2.537	73.881	0.0025	16.012	2.678	95.723	0.0025
Hypertension					2.152	0.503	9.205	0.2998	1.954	0.412	9.259	0.3968
Education years									0.933	0.723	1.203	0.5900
Having a caregiver									7.052	0.550	90.373	0.1326

For the mRS, (1) model I was a basic model adjusted for time and cognitive impairment; (2) model I plus NIHSS, premorbid disability, and hypertension (model II); and (3) model II plus education and having a caregiver (model III).

*NIHSS, National Institutes of Health Stroke Scale; mRS, modified Rankin Scale*.

**Figure 3:**
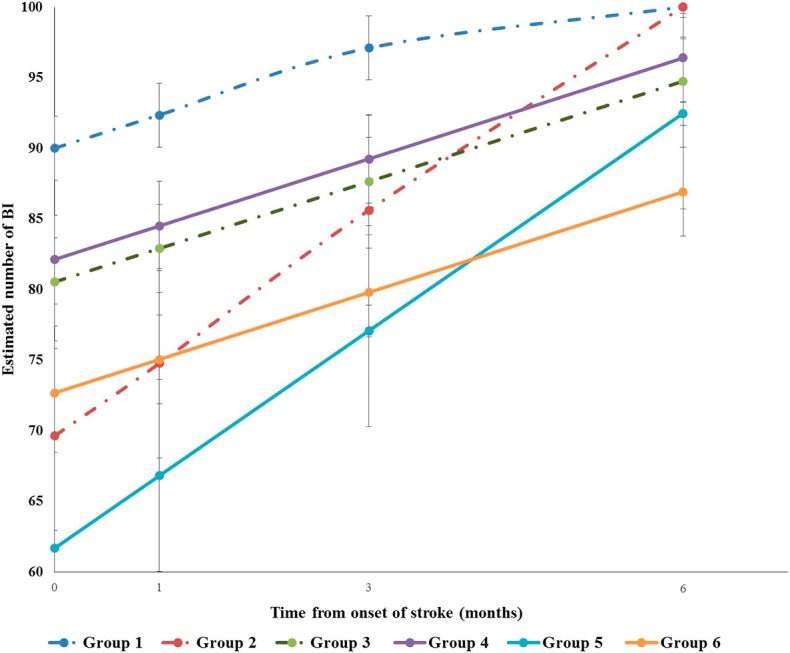
Trajectories of Barthel Index (BI) by baseline cognitive function, premorbid disability and rehabilitation. Group1 normal cognition without premorbid disability without rehabilitation in 1month after onset. Group2 normal cognition without premorbid disability with rehabilitation in 1month after onset. Group3 normal cognition with premorbid disability without rehabilitation in 1month after onset. Group4 cognition impairment without premorbid disability without rehabilitation in 1month after onset. Group5 cognition impairment without premorbid disability with rehabilitation in 1month after onset. Group6 cognition impairment with premorbid disability without rehabilitation in 1month after onset.

Table 4 presents the results from generalized linear mixed models of cognitive impairment and the covariates on BI. The BI score of all patients increased over time after controlling for the covariates (β = 1.51, *p* < 0.01), which meant that when time increased about 1 month, the BI score increased by 1.51 points. Cognitive impairment was significantly associated with BI. The BI score of those with cognitive impairment was lower than those with normal cognition over the follow-up period after controlling for other covariates (β = −8.11, *p* < 0.05). Patients who had higher NIHSS at admission had lower BI score at baseline (β = −1.65, *p* < 0.05) and showed a faster pace of BI rising over time than did those with lower NIHSS at admission (β = 0.27, *p* < 0.01). Subjects who received rehabilitation service in 1 month after onset had lower BI score at baseline (β = −21.31, *p* < 0.01) and showed a faster pace of BI rising over time than did those without rehabilitation service (β = 2.59, *p* < 0.01). Compared with those without premorbid disability, the patients with premorbid disability had lower score of BI (β = −7.07, *p* < 0.05). Subjects who had higher level of pain had lower BI score than had those with lower level of pain (β = −2.9591, *p* < 0.01). Figure 3 demonstrates the trajectories of BI by baseline cognitive function, premorbid disability, and rehabilitation after controlling for other covariates. The BI of Group 4 was lower than that of Group 1. The BI of Group 5 was lower than that of Group 2 with time. The BI of Group 6 was lower than that of Group 3 with time.

**Table 4:** The results from general linear mixed models of cognitive impairment and the covariates on BI.

**Variables**	**BI**											
	**Model I**				**Model II**				**Model III**			
	**Estimate**	**95% CI**		***p***	**Estimate**	**95% CI**		***p***	**Estimate**	**95% CI**		***p***
		**Lower**	**Upper**			**Lower**	**Upper**			**Lower**	**Upper**	
Intercept	85.2695	77.6462	92.8927	<0.0001	94.5132	85.8905	103.14	<0.0001	99.9090	90.7451	109.07	<0.0001
Time	3.1211	2.4040	3.8381	<0.0001	1.5323	0.5907	2.4739	0.0018	1.5125	0.5844	2.4407	0.0018
Cognitive impairment	−9.9874	−16.7709	−3.2039	0.0042	−9.2206	−16.8047	−1.6365	0.0176	−8.1067	−15.0944	−1.1190	0.0233
NIHSS (at admission)					−1.9630	−3.3060	−0.6200	0.0045	−1.6476	−2.9887	−0.3066	0.0164
NIHSS (admission) * time					0.3397	0.1400	0.5394	0.0010	0.2741	0.07145	0.4768	0.0084
Receiving rehabilitation					−24.1535	−36.7958	−11.5112	0.0002	−21.3089	−34.2312	−8.3866	0.0014
Receiving rehabilitation * time					2.8582	0.9182	4.7982	0.0042	2.5883	0.5979	4.5787	0.0112
Premorbid disability									−7.0704	−13.0672	−1.0736	0.0212
Hypertension									−5.8778	−12.0358	0.2802	0.0612
Pain (four time points)									−2.9591	−4.6123	−1.3060	0.0006
Pain * time									0.3858	−0.2206	0.9922	0.2104

For the BI, (1) model I was a basic model adjusted for time and cognitive impairment; (2) model I plus NIHSS, receiving rehabilitation 1 month after onset (model II); and (3) model II plus premorbid disability, hypertension, and pain (model III).

*BI, Barthel Index; NIHSS, National Institutes of Health Stroke Scale*.

Group 5, cognitive impairment without premorbid disability with rehabilitation in 1 month after onset. Group 6, cognitive impairment with premorbid disability without rehabilitation in 1 month after onset.

## Discussion

This study is the first longitudinal study to explore the trajectory of BI after the onset of stroke and to examine the association between early cognitive impairment screened by MoCA and midterm functional outcomes among Shanghai AIS patients. Functional outcomes (mRS and BI) were measured at each time point (0th, 1st, 3rd, and 6th month after stroke onset), in order to explore the trajectory of functional outcomes and the association with early cognitive impairment. Overall, the probability of disability decreased and the score of BI increased, indicating that the functional outcomes improved over the 6-month study period after controlling for covariates. Early cognitive impairment at acute stage after stroke was prospectively associated with poorer functional outcomes among Shanghai AIS patients after controlling for the sociodemographic characters and clinical characters. The patients with cognitive impairment at acute stage had lower level of independence in ADLs than had those with normal cognitive function from acute stage to all the follow-up periods. These findings add new evidence to the association between early cognitive impairment screened by MoCA after stroke and midterm functional outcomes among AIS patients. Our findings support that early cognitive impairment after stroke has been proven to be a predictor or determinant of midterm functional outcomes among Chinese AIS patients. They can provide health professionals useful information about the management and rehabilitation for stroke patients. They also support the clinical utility of early cognitive screening by MoCA after stroke. MoCA is a short instrument that can easily be fitted into the clinical schedule.

The result of the present study is consistent with those of previous longitudinal studies (14, 16, 36–39) in some other countries. These studies showed that cognitive impairment at acute stage and 3 and 18 months after stroke was associated with poor function and ADL on discharge, at 12 months, or at 3–4 years after stroke (14, 16, 36–39). The outcomes would suggest that cognitive impairment after stroke may be an important determinant of functional outcomes. However, these studies did not demonstrate the trajectories of functional outcomes after stroke onset, nor did they explore the association between early cognitive impairment after stroke and midterm disability and BI. Only one study reported the association between early MoCA and long-term cognitive and functional outcomes after stroke (16). Nevertheless, this study did not adjust the treatment options (intravenous thrombolysis and intra-arterial thrombectomy) when exploring the association between early MoCA and long-term function among stroke patients. Previous studies had demonstrated that the ischemic stroke patients who received intravenous thrombolysis treatment had milder aphasia than had those who received non-thrombolysis treatment at 1 week and 6 months after stroke (40), and patients who received mechanical thrombectomy had better neurological and cognitive functions at 6, 24 h, 7, and 90 days (41). In the present study, we have controlled the treatment options.

The mechanism between cognitive impairment after stroke and poor functional outcomes may be explained by the neuroanatomical lesions. Cognitive impairment after stroke often comprises a mix of vascular damage caused by stroke and neurodegenerative processes (42). Cognitive domains of cognitive impairment after stroke are associated with stroke type, volume, numbers, location, and severity (43). Important strategic locations include the medial frontal lobe, parietal lobe, angular gyrus, and the inferomedial portion of the temporal lobe, hippocampus, and thalamus. Frontal lobe functions comprising processing speed, reaction time, working memory, and executive task measures are most affected in post-stroke cognitive impairment (44, 45). Regardless of vascular damage or neurodegenerative process, these impaired cognitive functions are related to poor recovery outcomes (9, 46). These reasons may partially explain why the patients with cognitive impairment or dementia in the acute stage of stroke had poorer long-term outcomes, such as higher level of disability.

Many patients develop mild cognitive impairment after stroke. Some can even progress to post-stroke dementia (PSD) (46–48). Neuropsychologists have paid more attention to post-stroke cognitive impairment or dementia at 3–6 months or longer periods after stroke. However, little attention has been paid to early cognitive impairment in the acute phase of stroke. There is an urgent need to detect post-stroke cognitive impairment as early as possible. Screening for cognitive deficits was recommended for all stroke patients before discharge by the 2016 American Heart Association/American Stroke Association (AHA/ASA) guideline for Adult Stroke Rehabilitation and Recovery (Ib) (49). However, it was not included in the Chinese guidelines for diagnosis and treatment of AIS 2018 (50). The screening and detection of cognitive impairment in the acute stage of stroke can identify the patients who are at higher risk of having cognitive impairment and those who have developed cognitive impairment. It will also provide health-care professionals with vital information on planning early cognitive rehabilitation and developing a more person-centered follow-up plan (51). In that case, individuals with post-stroke cognitive impairment at acute stage may benefit from individualized stroke care and rehabilitation services and receive better information related to potential for recovery and quality of life in the long term. Controlling vascular risk factors and primary preventative strategies related to healthy lifestyle would not only help reduce the disease and caregiving burden of cognitive impairment and dementia after stroke but also potentially improve the patients' physical function and quality of life. This is supported by a study finding that using Neurobehavioral Cognitive Status Examination (NCSE) to assess the cognitive function of stroke patients before inpatient rehabilitation could help to predict their functional ability changes (52).

The strength of current study is that we explored the trajectories of midterm outcomes (mRS and BI) among stroke patients from acute stage to 6 months after onset. The scores of mRS and BI were measured for four time points and treated as time-varying variable, so that longitudinal analyses with generalized linear mixed models and generalized linear mixed models provide precise and robust estimates. Meanwhile, the significant association between early cognitive impairment and midterm outcomes of stroke patients was found. These outcomes support the recommendation for routine screening for cognitive impairment by MoCA among stroke patients early after hospital admission.

One limitation of the study was that the proportion of patients with low NIHSS score was high, because these patients were more likely to consent to participate in the study. It may underestimate NIHSS's predictive value in the models and overestimate MoCA's predictive value for disability and BI. Although we had included a small subgroup of patients with admission NIHSS score > 3, the conclusions of present study should be interpreted and generalized cautiously. Future studies with a larger representation of patients with major stroke are required to confirm the findings of this study. MoCA is the most widely used instrument for the screening of cognitive impairment. However, it is an assessment tool for global cognition, not a domain-specific tool. MoCA may have a lower specificity than the gold standard, which is a comprehensive neuropsychological battery (53). In further studies, neuropsychological test batteries should be used to assess all domains of cognition including attention and processing speed, executive function, learning and memory, language, visual-perceptual ability, praxis-gnosis-body schema, and social cognition (54). It would be helpful to increase the accuracy of diagnosis for post-stroke cognitive impairment and dementia and to examine the linkage between multi-domain cognitive impairment and long-term outcomes among stroke survivors. The last limitation was that the participants in present study might not be fully representative of stroke patients in China. Shanghai is the most developed area in China, and it has advanced health-care service system like that of developed countries. There are huge differences in health-care system and level of socioeconomic development across the regions in China. The different levels of health-care services in different areas may influence the recovery outcomes of stroke patients. The findings may not be generalizable to other areas of China. However, the conclusions may have implications for the developed countries.

## Conclusion

This is the first longitudinal study reporting the association between early cognitive impairment and midterm functional outcomes (mRS and BI) among Shanghai AIS patients. The results showed that early cognitive impairment at acute stage was prospectively associated with higher risk of disability among Shanghai AIS patients after controlling for all the covariates listed in Table 2. Early cognitive impairment was an independent predictor of midterm functional outcomes among stroke patients. The findings may provide useful information for clinicians to develop a special stroke treatment and early cognitive rehabilitation plan for patients, which would be beneficial to the improvement of long-term outcomes and quality of life of stroke patients. However, we understand that it is possible that the nature of the association between early cognitive impairment and midterm functional outcomes among AIS patients may be partially explained by the neuroanatomical lesions caused by stroke and neurodegenerative processes. In future studies, we will further examine the mechanisms that link early cognitive impairment and midterm physical outcomes and whether these linkages are mediated/moderated by other potential factors.

## Data Availability Statement

The data will not be shared due to Naval Military Medical University's regulations.

## Ethics Statement

The studies involving human participants were reviewed and approved by Institutional Review Board (IRB) of Second Military Medical University (2014LL001). The patients/participants provided their written informed consent to participate in this study.

## Author Contributions

JL designed the concept, collected, analyzed and interpreted data, and prepared the manuscript. JW and HX interpreted the outcome and reviewed the manuscript. BW and LZ designed the concept, interpreted the outcome, and reviewed the manuscript. XW collected the data. BD designed the concept and collected the data.

### Conflict of Interest

The authors declare that the research was conducted in the absence of any commercial or financial relationships that could be construed as a potential conflict of interest.
