# Supplementary material for: Association Between Early Cognitive Impairment and Midterm Functional Outcomes Among Chinese Acute Ischemic Stroke Patients: A Longitudinal Study
Source: Front Neurol. 2020 Feb 26;11:20. doi: 10.3389/fneur.2020.00020 (PMC7054458; doi:10.3389/fneur.2020.00020)
Supplement: Supplementary file 1 [file Table_1.DOCX]

Appendix 1

Table Baseline characteristics of patient samples from three stroke centers

| Characteristics | Center A (n=68) | Center B (n= 89 ) | Center C(n=28) | Pooled data | *P* Value |
| --- | --- | --- | --- | --- | --- |
| Age, y; median (IQR) | 61 (53-68) | 61 (57-65) | 62（56-68） | 61(56-66) | 0.790 |
| Sex, male(%) | 55 (80.9%) | 69(77.5%) | 23（82.1%） | 147(79.5%) | 0.814 |
| Ethnicity-Han | 68(100%) | 89(100%) | 28（100%） | 185（100%） |  |
| Education |  |  |  |  | 0.506 |
| Illiterate | 3 (4.4%) | 4 (4.5%) | 2（7.1%） | 9(4.9%) |  |
| Elementary school | 6 (8.8%) | 3 (3.4%) | 2（7.1%） | 11(5.9%) |  |
| Junior school | 27(39.7%) | 38 (42.7%) | 11（39.3%） | 76(41.1%) |  |
| High school | 18 (26.5%) | 35 (39.3%) | 9（32.1%） | 62(33.5%) |  |
| College and university | 14 (20.6%) | 9(10.1%) | 4（14.3%） | 27(14.6%) |  |
| Marital status, living spouse(%) | 63(92.6%) | 84 (94.4%) | 24（85.7%） | 171（92.4%） | 0.317 |
| Residency, Urban(%) | 62 (91.2%) | 80 (89.9%) | 28（100%） | 170（91.9%） | 0.223 |
| Working, yes(%) | 33 (48.5%) | 37 (41.6%) | 8（28.6%） | 78（42.2%） | 0.196 |
| Family income( Yuan/year） |  |  |  |  | 0.139 |
| ＜50,000 | 3 (10.0%) | 7 (23.3%) | 5（38.5%） | 38（20.5%） |  |
| 50,000-100,000 | 19 (63.3%) | 12 (40.0%) | 6（46.2%） | 94（50.8%） |  |
| >100,000 | 8 (26.7%) | 11 (36.7%) | 2（15.4%） | 53（28.7%） |  |
| NIHSS at admission, median (IQR) | 3 (1-5) | 1.5 (0-3) | 3（1-5） | 2（1-4） | 0.003* |
| NIHSS at discharge, median (IQR) | 1 (0-3) | 1 (0-2) | 2（1-4） | 1（0-3） | 0.004* |
| Dysphagia(%) | 19 (27.9%) | 26 (29.2%) | 11（39.3%） | 56(30.3%) | 0.522 |
| Pain, mean (SE) | 0.50 ( 1.91) | 0.70 (1.43) | 0.18（0.94） | 0.55 (1.57) | 0.301 |
| Length of stay, median (IQR) | 8.5 (8-9) | 7(6-7) | 11(9.25-13) | 8(7-9) | 0.000* |
| OCSP Classification |  |  |  |  | 0.262 |
| Total anterior circulation infarct(TACI) | 2 (3.2%) | 6(7.2%) | 0(0) | 9（4.7%） |  |
| Partial anterior circulation infarct(PACI) | 26 (41.3%) | 28 (33.7%) | 14(58.3%) | 74（40.0%） |  |
| Posterior circulation infarct(POCI) | 22 (34.9%) | 25(30.1%) | 6(25%) | 58（31.2%） |  |
| Lacunar circulation infarcts(LACI) | 13 (20.6%) | 24 (28.9%) | 4(16.7%) | 44（24.1%） |  |
| TOAST Classification |  |  |  |  | 0.006* |
| large-artery atherothrombotic (LAA) | 32 (47.1%) | 32 (36.0%) | 21(75%) | 85（45.9%） |  |
| cardioembolic (CE) | 8 (11.8%) | 4 (4.5%) | 1(3.6%) | 13（7%） |  |
| small-artery occlusion (SAO) | 24(35.3%) | 38 (42.7%) | 6(21.4%) | 68（36.8%） |  |
| other determined etiology(ODE) | 3 (4.4%) | 8 (9%) | 0(0) | 11（5.9%） |  |
| Undetermined etiology(UDE) | 1 (1.5%) | 7 (7.9%) | 0(0) | 8（4.3%） |  |
| Intravenous thrombolysis(%) | 13 (19.1%) | 9 (10.1%) | 1(3.6%) | 23（12.4%） | 0.072 |
| Intra-arterial thrombectomy(%) | 2 (2.9%) | 1 (1.1%) | 0(0) | 3（1.6%） | 0.511 |
| onset to door time |  |  |  |  | 0.361 |
| <1 hour | 14 (20.6%) | 21 (23.6%) | 10(35.7%) | 45（24.3%） |  |
| 1-3 hour | 19 (27.9%) | 17 (19.1%) | 2(7.1%) | 38（20.5%） |  |
| 3.1-4.5 hour | 3 (4.4%) | 3 (3.4%) | 0(0) | 6（3.2%） |  |
| 4.6-6 hour | 4 (5.9%) | 6 (6.7%) | 1(3.6%) | 11（5.9%） |  |
| >6 hour | 20 (29.4%) | 35 (39.3%) | 10(35.7%) | 65（35.1%） |  |
| unclear | 8 (11.8%) | 7 (7.9%) | 5(17.9%) | 20（10.8%） |  |
| Comorbidities |  |  |  |  |  |
| hypertension | 52 (76.5%) | 60(67.4%) | 20(71.4%) | 132（71.4%） | 0.462 |
| Diabetes | 29 (42.6%) | 26(29.2%) | 16(57.1%) | 71（38.4%） | 0.020* |
| dyslipidemia | 35 (51.5%) | 10(11.2%) | 11(39.3%) | 56（30.3%） | 0.000* |
| obstructive sleep apnea syndrome | 1 (1.5%) | 0 (0) | 1(3.6%) | 2（1.1%） | 0.260 |
| carotid atherosclerosis | 5(7.4%) | 2 (2.2%) | 1(3.6%) | 8（4.3%） | 0.290 |
| atrial fibrillation | 8(11.8%) | 4(4.5%) | 1(3.6%) | 13（7%） | 0.156 |
| Others | 1 (1.5) | 4(4.5%) | 0(0) | 5（2.7%） | 0.324 |
| BMI |  |  |  |  | 0.050 |
| Low weight (<18.5) | 0 (0) | 1 (1.4%) | 2(9.1%) | 3（1.6%） |  |
| Normal (18.5-23.9) | 23(35.4%) | 34 (45.9%) | 9(40.9%) | 76（41.1%） |  |
| Overweight (24-27.9) | 33 (50.8%) | 36 (48.6%) | 9(40.9%) | 90（48.7%） |  |
| Obesity(≥28) | 9 (13.8%) | 3 (4.1%) | 2(9.1%) | 16（8.6%） |  |
| SBP, mmHg | 138(130, 150) | 150(138, 160) | 147(134,173) | 144(132,160) | 0.005* |
| DBP, mmHg | 80(80, 92) | 85(79, 94) | 90(80,100) | 84(80,94) | 0.082 |
| Biochemical measurement |  |  |  |  |  |
| Fasting glucose, mg/dL | 5.7(5.1, 7.4) | 5.4(4.8, 6.8) | 6.8(5.2,7.9) | 5.6(5,7.3) | 0.100 |
| LDL-C, mg/dL | 2.6(2.1, 3.1) | 2.6(2.2, 3.2) | 3.1(2.4,4.4) | 2.6(2.2,3.2) | 0.007* |
| HDL-C, mg/dL | 1.0(0.8, 1.2) | 1.0(0.8, 1.3) | 1.0(0.9-1.3) | 1.0(0.9,1.3) | 0.297 |
| Triglycerides, mg/dL | 1.3(1.0, 2.1) | 1.5(1.0, 1.9) | 1.4(1.1,2.0) | 1.4(1.1,2.0) | 0.869 |
| Family history of ICH(%) | 19(27.9%) | 9(10.1%) | 2(7.1%) | 30（16.2%） | 0.004* |
| Family history of AIS(%) | 21(30.9%) | 25 (28.1%) | 10(35.7%) | 56（30.3%） | 0.739 |
| Previous stroke history(%) | 11 (16.2%) | 15 (16.9%) | 7(25.0%) | 33(17.8%) | 0.558 |
| Intracranial vascular stenosis(%) |  |  |  |  | 0.000* |
| No | 1 (1.9%) | 41(53.9%) | 4(14.8%) | 46(29.7%) |  |
| Mild | 41 (78.8%) | 10 (13.2%) | 13(48.1%) | 64(41.3%) |  |
| Moderate | 5 (9.6%) | 10(25.0%) | 9(33.3%) | 33(21.3%) |  |
| Severe | 5 (9.6%) | 6 (7.9%) | 1(3.7%) | 12(7.7%) |  |
| Smoking(%) | 34 (50%) | 45(50.6%) | 17(60.7%) | 96（51.9%） | 0.596 |
| Drinking(%) | 37 (54.4%) | 44 (49.4%) | 17(60.7) | 98（53.0%） | 0.555 |
| Premorbid disability | 20 (30.3%) | 5 (5.7%) | 3(10.7%) | 28(15.5%) | 0.000* |
| receiving rehabilitation in 1 month after onset | 8 (11.9%） | 2 (2.4%) | 1(3.6%) | 11(6.1%) | 0.043* |
| recurrence in 1 month after onset | 1(1.5%) | 2 (2.2%) | 1(3.6%) | 4(2.2%) | 0.796 |
| recurrence in 3 month after onset | 2(2.9%) | 2 (2.2%) | 0(0) | 4(2.2%) | 0.555 |
| recurrence in 6 month after onset | 0 (0) | 1 (1.1%) | 0(0) | 1(0.5%) | 0.578 |

TACI, Total anterior circulation infarct; PACI, Partial anterior circulation infarct; POCI, Posterior circulation infarct; LACI, Lacunar circulation infarcts; LAA, large-artery atherothrombotic; CE, cardioembolic; SAO, small-artery occlusion; ODE, other determined etiology; UDE, Undetermined etiology.
